# Identification and Expression Profiling of Chemosensory Genes in *Hermetia illucens* via a Transcriptomic Analysis

**DOI:** 10.3389/fphys.2020.00720

**Published:** 2020-06-19

**Authors:** Qiyun Xu, Zhongzhen Wu, Xinnian Zeng, Xincheng An

**Affiliations:** ^1^Guangdong Engineering Research Center for Insect Behavior Regulation, College of Agriculture, South China Agricultural University, Guangzhou, China; ^2^Guangdong Key Laboratory of Animal Conservation and Resource Utilization, Guangdong Institute of Applied Biological Resources, Guangzhou, China; ^3^Guangzhou City Key Laboratory of Subtropical Fruit Trees Outbreak Control, Zhongkai University of Agriculture and Engineering, Guangzhou, China

**Keywords:** antennal transcriptome, chemosensory gene, identification, expression analysis, *Hermetia illucens*

## Abstract

The black soldier fly, *Hermetia illucens*, is a cosmopolitan insect of the family Stratiomyidae (Diptera). Chemosensory genes encode proteins involved directly in the detection of odorants. In this study, we sequenced the antennal transcriptome of *H. illucens* adults to identify chemosensory genes. Putative unigenes encoding 27 odorant binding proteins (OBPs), five chemosensory proteins (CSPs), 70 odorant receptors (ORs), 25 ionotropic receptors (IRs), 10 gustatory receptors (GRs) and two sensory neuron membrane proteins (SNMPs) were identified. Tissue-specific expression profiles of the identified OBPs, CSPs and SNMPs were investigated using RT-PCR. Eight OBPs (*HillOBP1-2*, *9*, *11-14*, and *17*), one CSP (*HillCSP5*) and one SNMP (*HillSNMP1*) were predominantly expressed in antennae. Further real-time quantitative PCR analyses revealed that the antennae-enriched unigenes also exhibited significant differences in expression between males and females. Among the sex-biased unigenes, six ORs showed female-biased expression, suggesting that these genes might participate in female-specific behaviors such as oviposition site searching. Sixteen ORs and two OBPs showed male-biased expression, indicating that they may play key roles in the detection of female sex pheromones. Our study is the first attempt to delineate the molecular basis of chemoreception in *H. illucens*. Our data provide useful information for comparative studies on the differentiation and evolution of Dipteran chemosensory gene families.

## Introduction

Olfaction plays a crucial role in insect behaviors, such as foraging, mating, oviposition and avoiding predators (Leal, 2013). The process of olfactory detection is mediated by a number of gene families including odorant-binding proteins (OBPs), chemosensory proteins (CSPs), sensory neuron membrane proteins (SNMPs), and olfactory receptors (ORs), ionotropic receptors (IRs) and gustatory receptors (GRs) (Clyne et al., 1999, 2000; Vosshall et al., 1999; Galindo and Smith, 2001; Benton et al., 2009; Vieira and Rozas, 2011). Each group of proteins participates in different steps of the chemosensory process. OBPs and CSPs are thought to bind, solubilize and transport hydrophobic odorants across the aqueous lymph surrounding the olfactory sensory neurons (OSNs) on the sensilla of antennae (Wojtasek and Leal, 1999; Sandler et al., 2000; Xu et al., 2005; Gomez-Diaz et al., 2013). ORs are responsible for the detection of odorants, whereas IRs are involved in sensing chemo-, thermo- and hygro-sensory stimuli (Ai et al., 2010, 2013; Grosjean et al., 2011; Silbering et al., 2011; Kain et al., 2013; Su and Carlson, 2013; Koh et al., 2014; Chen et al., 2015; Stewart et al., 2015; Gorter et al., 2016; Hussain et al., 2016; Knecht et al., 2016, 2017; Ni et al., 2016). GRs are thought to be involved in the detection of sugars, bitter tasting compounds and non-volatile pheromones (Clyne et al., 2000; Bray and Amrein, 2003; Dahanukar et al., 2007; Jiao et al., 2007; Sung et al., 2017) and carbon dioxide (Jones et al., 2007; Turner and Ray, 2009; Tauxe et al., 2013). SNMPs may play important roles in pheromone sensing based on their expression on the dendritic membrane of pheromone sensitive neurons (Benton et al., 2007; Gomez-Diaz et al., 2016).

After the comprehensive characterization of chemosensory genes in the two model species *Drosophila melanogaster* and *Anopheles gambiae* (Robertson et al., 2003; Rinker et al., 2013) a growing number of chemosensory genes have also been identified from many other Dipteran species based on sequence similarity. These Dipterans include *Musca domestica* (Scott et al., 2014) *Bactrocera dorsalis* (Wu et al., 2015; Jin et al., 2017), *Calliphora stygia* (Leitch et al., 2015), *Glossina morsitans morsitans* (Macharia et al., 2016), *Mayetiola destructor* Say (Andersson et al., 2014), *Episyrphus balteatus* and *Eupeodes corollae* (Wang et al., 2017), and *Chlorops oryzae* (Qiu et al., 2018).

The black soldier fly, *Hermetia illucens*, is a cosmopolitan Dipteran. The larvae of *H. illucens* play a pivotal role in terms of both environmental and economic aspects for waste disposal and processing. *H. illucens* larvae are useful in manure management for controlling housefly populations and converting organic waste into useful products such as compost (Newton et al., 2005). Like other many Dipteran species, *H. illucens* adults have a sensitive olfactory system and use a wide range of environmental chemical cues to locate food, mates, and egg-laying sites (Booth and Sheppard, 1984; Tomberlin and Sheppard, 2001, 2002). *H. illucens* could potentially be used to decompose landfills if adult flies can be guided to deposit their eggs there. A better understanding on the molecular components of the *H. illucens* olfactory system is an initial step toward this type of practical application. Identification and characterization of chemosensory genes are also important steps toward understanding their evolution and primary functions. The objective of this study is to identify candidate chemosensory genes encoding OBPs, CSPs, ORs, IRs, GRs, and SNMPs by generating and analyzing the antennal transcriptome of *H. illucens* adults. Genetic and phylogenetic analyses as well as expression profiling of identified chemosensory genes were also carried out to gain insights on their potential functions.

## Materials and Methods

### Insects, Tissues Collection and RNA Isolation

*Hermetia illucens* adults were obtained from a colony maintained year-round in the laboratory of the Guangdong Public Laboratory of Wild Animal Conservation and Utilization, Guangdong Institute of Applied Biological Resources in Guangzhou City, Guangdong Province, China. The colony has been maintained at 28°C with a photoperiod of 14:10 h (Light: Dark) and 70% relative humidity.

For transcriptomic analyses, 150 pairs of antennae were collected separately from both females and males of *H. illucens* adults. For RT-PCR analysis, 50 tissues of antennae, mouthparts, foreleg tarsus, wings and genitals were separately obtained from adult males and females. Three replicates were generated for each tissue set.

Total RNA was isolated from homogenized tissues using Trizol reagent (Invitrogen, Carlsbad, CA, United States) following the manufacturer’s instructions and then treated with RNase-free DNase I (TaKaRa, Dalian, China) to remove potential genomic DNA contamination. RNA integrity was monitored on 1% agarose gel, and assessed with an Agilent 2100 Bioanalyzer (Agilent Technologies, Santa Clara, CA, United States). RNA concentration and purity were analyzed on a NanoDrop ND-2000 Spectrophotometer (Nanodrop Technologies, Wilmington, DE, United States).

### cDNA Library Construction, Sequencing and *de novo* Assembly

cDNA libraries were constructed with 1.5 μg purified RNA using a TruSeq RNA Sample Preparation Kit (Illumina, San Diego, CA, United States) following the manufacturer’s instruction. Library preparations were sequenced using the Illumina HiseqTM 2500 platform (San Diego, CA, United States) and paired-end reads were generated. After sequencing, raw reads were firstly processed through in-house perl scripts. Clean reads were obtained from raw data after removing reads containing adapter, unknown (poly-N) and low-quality reads. Clean reads assembly was accomplished using Trinity (Version: r2013-11-10) with the default parameters after combining the male and female clean reads (Grabherr et al., 2011). The largest assembly sequences were deemed to be unigenes. The clean reads from the antennal transcriptome of *H. illucens* were uploaded to the NCBI Sequence Read Archive (SAMN12915779).

### Functional Annotation

BLASTx searches were carried out against sequences in the NCBI non-redundant protein (nr) protein database with a cut-of *E*-value of 10^–5^. Unigenes were also annotated using other databases including NCBI non-redundant nucleotide (nt), Swiss-Prot, the Kyoto Encyclopedia of Genes and Genomes (KEGG), Gene Orthology (GO) and Cluster of Orthologous Groups of proteins (COG).

### Gene Identification

To identify chemosensory genes from *H. illucens*, known OBPs, CSPs, ORs, IRs, GRs, and SNMPs from other Dipteran insects were selected as queries to search the *H. illucens* antennal transcriptome. Query OBPs were from *D. melanogaster*, *B. dorsalis*, *Ceratitis capitata*, and *M. domestica*. Query CSPs were from *D. melanogaster*, *B. dorsalis*, *M. domestica* and *G. morsitans*. Query ORs were from *D. melanogaster*, *B. dorsalis*, *C. stygia*, and *M. domestica*. Query GRs were from *D. melanogaster*, *C. stygia* and *C. capitata* for IRs; *D. melanogaster*, *B. dorsalis* and *C. stygia*. Query SNMPs were from *D. melanogaster*, *B. dorsalis*, and *M. domestica* (Supplementary Table S1). tBLASTn was used to identify candidate unigenes encoding OBPs, CSPs, ORs, IRs, GRs, and SNMPs against the *H. illucens* antennal transcriptome with a cut-of *E*-value of 10^–5^. Identified candidate unigenes were manually checked using BLASTx against the NCBI non-redundant protein sequences database. Potential open reading frames (ORFs) of candidate chemosensory genes were predicted using the NCBI ORF Finder^1^. Alignments of amino acid sequences were performed using MAFFT (Version: 7.308) (E-INS-I parameter set) (Katoh and Standley, 2013) and visualized with Geneious (Version: 9.1.3) (Kearse et al., 2012). Protein domains (including transmembrane domains and signal peptides) were predicted using the InterProScan tool plug-in in Geneious (Quevillon et al., 2005). tBLASTn searches were used to determine the scaffold location, position and intron-exon organization of the candidate chemosensory genes in each *H. illucens*’s genomic scaffold (GCA_009835165.1). Candidate unigenes coding for chemosensory genes and their corresponding reference genes were listed in Supplementary Table S2.

### Phylogenetic Analysis

Amino acid sequences of candidate OBPs, CSPs, ORs, IRs, GRs, and SNMPs from *H. illucens* were aligned together with proteins from other Dipterans. The sequences from other Dipterans used for building phylogenetic trees are listed in Supplementary Table S3. Sequence alignments were generated using Clustal Omega (Sievers et al., 2011), and maximum-likelihood trees were constructed using FastTree2 (Jones-Taylor-Thornton amino acid substitution model) with default settings and 1000 bootstrap replicates (Price et al., 2010). Phylogenetic trees were colored and arranged using FigTree (Version: 1.4.2).

### Analyses of Expression Levels Based on FPKM

Clean reads were mapped back onto the assembled unigenes and read count for each unigene was obtained from the mapping results. The expression levels of these unigenes were calculated as the fragments per kilobase per million mapped fragments (FPKM) method (Trapnell et al., 2010).

### RT-PCR and Real-Time Quantitative PCR

The expression profiles of 27 OBPs, 5 CSPs, and 2 SNMPs among various tissues (antennae, mouthparts, foreleg tarsus, wings, and genitals) were initially evaluated using RT-PCR. Total RNA was isolated from these tissues, and cDNA was synthesized using a PrimeScript RT reagent Kit (Takara). In our pre-experiment, α-tubulin (*α-TUB*) and glyceraldehyde-3-phosphate dehydrogenase (*GAPDH*) were evaluated as the most stable reference genes for gene expression profiling across the different tissues (male antennae, female antennae mouthparts, foreleg tarsus, wings, and genitals) using Normfinder (Andersen et al., 2004) and BestKeeper (Pfaffl et al., 2004) (Supplementary Table S4). Therefore, *α-TUB* and *GAPDH* genes were used as controls to assess the cDNA integrity. PCR reactions were conducted using a Bio-Rad thermal cycler (Bio-Rad, Hercules, CA, United States) with the same cycling parameters as our previous study (Cui et al., 2019). PCR products were analyzed by electrophoresis in 1.5% agarose gels. Each PCR reaction was repeated twice with independently isolated RNA samples.

Based on the RT-PCR results, unigenes encoding OBPs, CSPs, IRs, and ORs that were predominantly or exclusively expressed in antennae were analyzed again for more accurate estimation using real-time quantitative PCR (RT-qPCR). RT-qPCR analysis was conducted using a LightCycler 480 system (Roche Applied Science, Basel, Switzerland). PCR reaction conditions were the same to those used in our previous study (Cui et al., 2019). Each RT-qPCR reaction was performed in three technical replications with three independent biological replications, and PCRs with no template (nuclease-free water) were used as negative controls. RT-qPCR analysis was performed using the LightCycler 480 gene scanning software. Relative gene expression level was quantified using the comparative 2^–ΔΔCT^ method (Livak and Schmittgen, 2001) and calculated relative to *α-TUB* and *GAPDH*. All gene-specific primers were designed using Primer3 (Version: 4.1.0)^2^ (Supplementary Table S5).

### Statistical Analysis

Data analysis was conducted using SPSS 22.0 (SPSS Inc., Chicago, IL, United States). The significant difference analysis of each gene among various tissues was determined using a one-way nested analysis of variance (ANOVA), following by Duncan’s new multiple range test (*α* = 0.05). Values are presented as mean ± SE. The GraphPad Prism 6.0 software (GraphPad Inc, San Diego) was used to perform the figures.

## Results

### Transcriptome Sequencing and *de novo* Assembly

In total, 118.56 million raw reads (17.78 GB raw data) were obtained from the antennal transcriptome of *H. illucens*. Additionally, 117.99 million of clean reads (17.70 GB clean data) were generated after filtering adapters and low-quality raw sequences. The clean reads were assembled into 70,124 unigenes with an N50 of 2,102 bp, average length of 1,002 bp (Supplementary Table S6). Length distribution analysis indicated 33,523 unigenes, which accounted for 47.8% of all unigenes, were longer than 500 bp (Supplementary Table S7).

### Functional Annotation

There were 24,837 (35.42%), 11,836 (16.88%), 16,633 (23.72%), 7,051 (10.06%), 7,873 (11.23%), and 17,675 (25.21%) unigenes that had homologous sequences in NCBI-nr, NCBI-nt, Swiss-Prot, GO, COG and KEGG databases, respectively. In total, 26,259 (37.45%) unigenes were annotated and the remaining unigenes were unmappable at present based on the sequence homology (Supplementary Figure S1A). Low homology with other insect species in NCBI was observed. The highest match percentage (6.84%) was identified with sequences of *Lucilia cuprina* followed by sequences of *M. domestica* (5.68%) and *C. capitata* (5.65%) (Supplementary Figure S1B). Gene ontology (GO) analysis was used to classify unigenes into different functional categories (Supplementary Figure S1C). In the ‘biological process’ category, the subcategories “cellular,” “metabolic” and “single-organism” process were the most represented. In the ‘cellular component’ category, the subcategories “cell” and “cell part” and “membrane” were the most represented. In the ‘molecular function’ category, the subcategories “binding” and “catalytic activity” were most represented.

### Transcript Abundance in *H. illucens* Antennae

The expression levels of all unigenes are given in Supplementary Table S8. Unigenes with FPKM values ≥ 1,000 were defined as highly expressed, those with FPKM 200∼1,000 were defined as moderately expressed, and those with FPKM ≤ 200 were defined as weakly expressed. According to these criteria, two unigenes coding for CSPs (named *HillCSP1* and *2*) and three unigenes coding for OBPs (named *HillOBP1*, *2*, and *3*) were abundant in antennae with 24,339, 2,654, 5,664, 5,368, and 4,918 FPKM, respectively, suggesting their potential roles in odorant detection. Other highly abundant unigenes included those coding for the cytochrome oxidase subunit 2 (mitochondrion) (CL10816.Contig1_All, 4,103 FPKM), the cytochrome oxidase subunit 1 (mitochondrion) (CL14318.Contig1_All, 2,942 FPKM), and cytochrome oxidase subunit 3 (mitochondrion) (Unigene12013_All, 2,908 FPKM). Interestingly, a unigene encoding a takeout-like protein was also highly expressed in *H. illucens* antennae (Unigene14734_All, 2,446 FPKM).

### Candidate Odorant Binding Proteins (OBPs)

**FIGURE 1:**
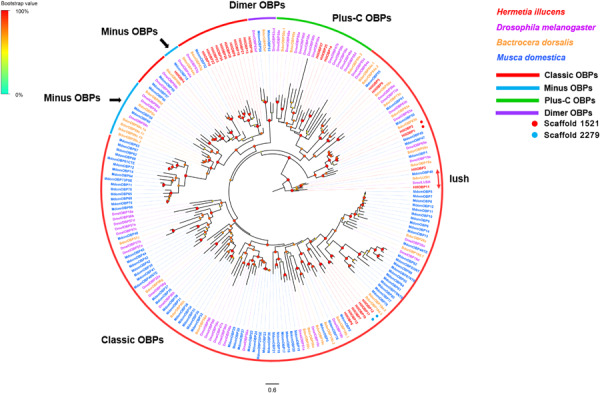
Maximum likelihood tree of candidate OBPs from *H. illucens* and other Dipteran insects. The distance tree was rooted by the lush orthologs. Branch support was estimated using 1000 bootstrap replicates, and bootstrap values were displayed with color circles at the branch nodes. Bars indicate the expected number of amino acid substitutions per site. Classic OBPs are in red; Minus OBPs in blue; Plus-C OBPs in green; Dimer OBPs in purple. OBPs located in the scaffold 1521 are highlighted with red dots. OBPs located in the scaffold 2279 are highlighted with blue dots.

Twenty-seven OBP-encoding unigenes (*HillOBP1-27*) were identified from the *H. illucens* antennal transcriptome (Supplementary Table S2: Sheet 1). The 27 *H. illucens* OBPs were distributed among 16 different scaffolds, with *HillOBP8/20/21/22/23/24/26* collocated in scaffold 2649, *HillOBP4/10/16* collocated in scaffold 1747, *HillOBP1/2* located in scaffold 1521, *HillOBP3/15* located in scaffold 2041, *HillOBP12/17* located in scaffold 2279. All identified OBP unigenes except three (*HillOBP5*, *21* and *22*) had a full-length ORFs encoding proteins with 130 to 233 amino acid residues. All these predicted proteins have a putative signal peptide at the N terminal region. Sequence identities of predicted OBPs with those from other Dipterans in the NCBI-nr database ranged from 26.55 to 76.55%, with an average of 39.48%. According to the number and location of the conserved cysteines (Hekmat-Scafe et al., 2002), twenty four OBPs (*HillOBP1-4*, *6-20*, *23-27*) were classified as classic OBPs, with the typical six conserved C-residues (Supplementary Figure S2), and three OBPs (*HillOBP14*, *25*, and *27*) were classified as plus-C OBPs, with four to six additional cysteine residues in addition to the six conserved cysteine residues. Phylogenetic analyses of OBPs from *H. illucens* and other three Dipteran species (*D. melanogaster*, *B. dorsalis*, and *M. domestica*) showed that all the *H. illucens* OBPs formed distinct clades based on an insect OBP classification system, and segregated into the classic OBP and plus-C OBP sub-families (Figure 1). FPKM value analysis revealed that five OBPs (*HillOBP1-5*) were highly expressed in *H. illucens* antennae (FPKM >1,000) (Supplementary Table S2: Sheet 1).

### Candidate Chemosensory Proteins (CSPs)

**FIGURE 2:**
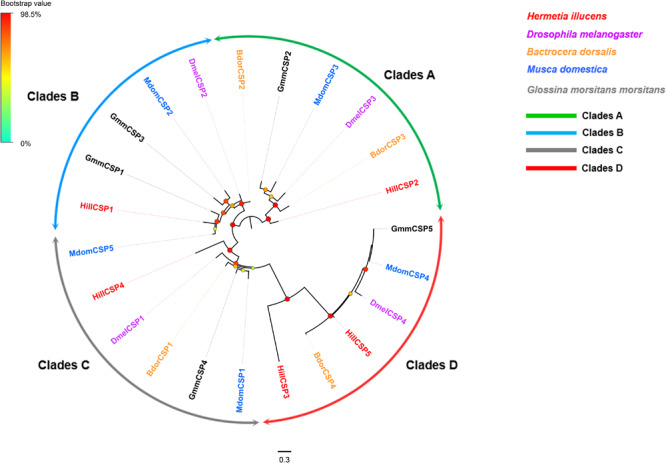
Maximum likelihood tree of candidate CSPs from *H. illucens* and other Dipteran insects. Branch support was estimated using 1000 bootstrap replicates, and bootstrap values were displayed with color circles at the branch nodes. The scale bar indicate the expected number of amino acid substitutions per site. Clades A are in green; Clades B in blue; Clades C in gray; Clades D in red.

Five CSP-encoding unigenes (*HillCSP1-5*) were identified (Supplementary Table S2: Sheet 2). The five *H. illucens* CSPs were distributed among two different scaffolds, with *HillCSP1/2/4/5* collocated in scaffold 1059, *HillCSP3* located in scaffold 1518. All the unigenes encoding CSPs have full-length ORFs encoding proteins with 110–128 amino acid residues, including four highly conserved cysteine residues and a signal peptide (Supplementary Figure S3). A phylogenetic tree was constructed using all predicted *HillCSPs* together with those from other Dipteran species. The CSPs of *H. illucens* formed four distinct clades marked as A, B, C, and D in Figure 2. Based on FPKM values, *HillCSP1* is the most highly expressed in antennae with FPKM >24,000, followed by *HillCSP2* (FPKM >2,000) (Supplementary Table S2: Sheet 2).

### Candidate Odorant Receptors (ORs)

**FIGURE 3:**
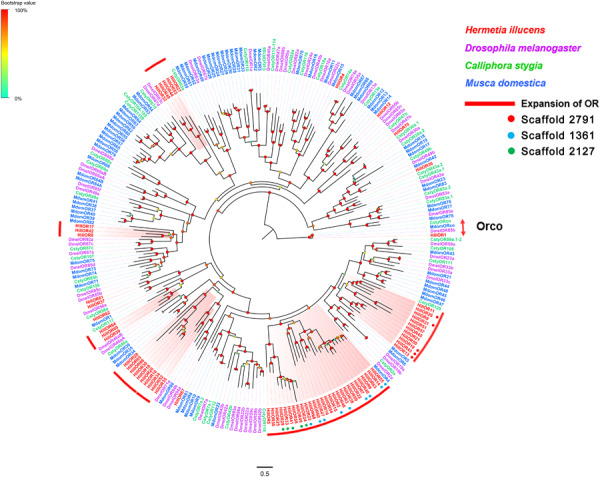
Maximum likelihood tree of candidate ORs from *H. illucens* and other Dipteran insects. The distance tree was rooted by the conservative ORco orthologs. The species-specific clades are labeled with red. Branch support was estimated using 1000 bootstrap replicates, and bootstrap values were displayed with color circles at the branch nodes. The scale bar indicate the expected number of amino acid substitutions per site. ORs located in the scaffold 2791 are highlighted with red dots. ORs located in the scaffold 1361 are highlighted with blue dots. ORs located in the scaffold 2171 are highlighted with green dots.

Seventy OR-encoding unigenes were identified, including one Orco (*HillOR1*) and 69 conventional OR genes (*HillOR2-70*) (Supplementary Table S2: Sheet 3). The 70 *H. illucens* ORs were distributed among 38 different scaffolds, with *HillOR62/69* located in scaffold 1002, *HillOR6/38/63* collocated in scaffold 1069, *HillOR10/18/20/30* collocated in scaffold 1106, *HillOR32/44/53* collocated in scaffold 1295, *HillOR2/3/7/14/23/40/47/55/60* collocated in scaffold 1361, *HillOR51/57* collocated in scaffold 1950, *HillOR24/29/33/41/49* collocated in scaffold 2127, *HillOR15/54* collocated in scaffold 2245, *HillOR5/25/58* collocated in scaffold 2284, *HillOR13/19/26/31/43* collocated in scaffold 2791, *HillOR37/61* located in scaffold 281, *HillOR21/50* located in scaffold 300, *HillOR39/64* located in scaffold 440, *HillOR35/52* located in scaffold 793, *HillOR22/59* located in scaffold 914. Among these OR unigenes, 48 have full-length ORFs encoding proteins with 360 to 477 amino acid residues. Four of the predicted proteins have 4–8 transmembrane domains (TMDs). The highly conserved co-receptor *HillOR1* shared 82.53% identity with a co-receptor from *C. capitata* (XP_012156143), while other *HillORs* shared 21.51–82.53% identity with those from other Dipterans. A phylogenetic tree was constructed using our identified ORs along with a data set containing representative ORs from three other Dipterans, including *D. melanogaster*, *C. stygia* and *M. domestica* (Figure 3). The vast majority of *HillORs* formed several species-specific clades, and no ORs in *H. illucens* clustered with orthologs from other species. Among these *HillORs*, *HillOrco* had the highest expressional level (FPKM = 1,159.56), whereas the other *HillORs* are weakly expressed (FPKM: 1.12∼140.25) (Supplementary Table S2: Sheet 3).

### Candidate Ionotropic Receptors (iGluRs/IRs)

**FIGURE 4:**
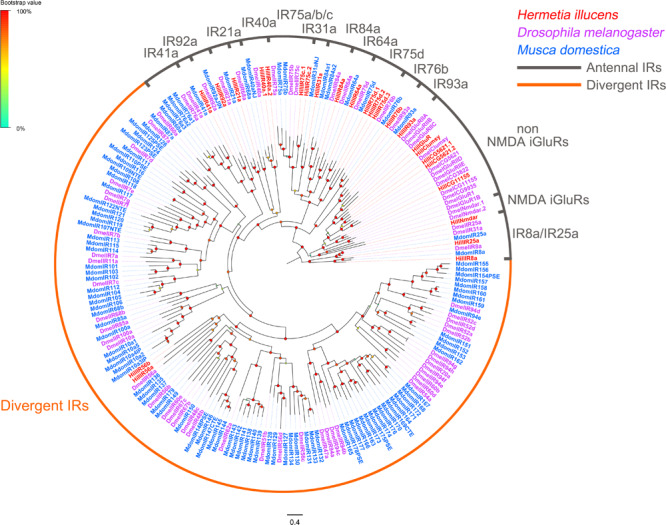
Maximum likelihood of candidate IRs from *H. illucens* and other Dipteran insects. The distance tree was rooted by the conservative IR8a/IR25a orthologs. Branch support was estimated using 1000 bootstrap replicates, and bootstrap values were displayed with color circles at the branch nodes. The scale bar indicate the expected number of amino acid substitutions per site. Antennal IRs are in gray; Divergent IRs are in orange.

Twenty-five iGluR/IR-encoding unigenes were identified (Supplementary Table S2: Sheet 4). Of these iGluR/IR unigenes, twenty have full-length ORFs encoding proteins with at least 426 amino acid residues. The 25 *H. illucens* iGluR/IR were distributed among 21 different scaffolds, with *HillCG5621.1/CG5621.2/IR93a* collocated in scaffold 2289, *HillIR75c.1/75c.2* located in scaffold 435 and *HillIR40a.1/40a.2* located in scaffold 2311. Distinct clades were observed in a phylogenetic tree constructed with our identified iGluRs/IRs and orthologs from *D. melanogaster* and *M. domestica* (Figure 4). Among the identified iGluR/IRs, 17 antennal IRs clustered with previously reported “antennal” orthologs *HillIR8a, 25a*, *21a*, *40a.1*, *40a.2*, *41a*, *64a*, *75c.1*, *75c.2*, *75d.1*, *75d.2*, *75d.3*, *76b*, *84a*, *92a*, and *93a*; and were clearly separated from those non-NMDA iGluRs, NMDA iGluRs and divergent IRs clades. Interestingly, a usually conserved “antennal” ortholog, *IR76a*, was absent from *H. illucens*. Instead, two IR75c orthologs (*IR75c.1* and *75c.2*), and three IR75d orthologs (*IR75d.1*, *75d.2*, and *75d.3*) was found in *H. illucens.* FPKM value analysis indicated that all these iGluR/IR unigenes were expressed at very low levels (the average FPKM value of 7.55), and only three “IR co-receptor” orthologs *(HillIR25a, 8a*, and *76b*) were expressed at relatively higher levels (FPKM >20).

### Candidate Gustatory Receptors (GRs)

**FIGURE 5:**
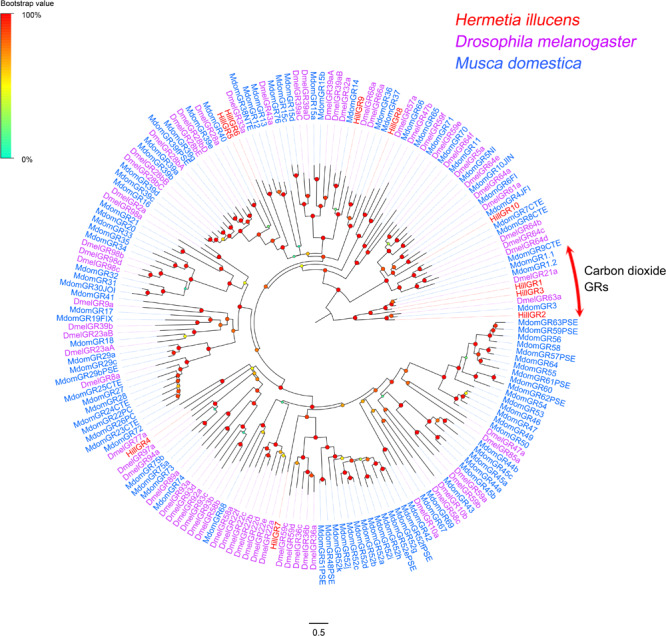
Maximum likelihood of candidate GRs from *H. illucens* and other Dipteran insects. The distance tree was rooted by the conservative carbon dioxide GRs orthologs. Branch support was estimated using 1000 bootstrap replicates, and bootstrap values were displayed with color circles at the branch nodes. The scale bar indicate the expected number of amino acid substitutions per site.

Ten GR-encoding unigenes were identified, and four of them encode full-length proteins with 6–8 TMDs (Supplementary Table S2: Sheet 5). The 10 *H. illucens* GRs were distributed among 10 different scaffolds. Potential functions of GRs identified from *H. illucens* could be inferred from their phylogenetic relationship with GRs previously well characterized from other Dipteran species (Figure 5). *HillGR1*, *2*, and *3* were clustered with carbon dioxide GRs (*DmelGR21a* and *63a*) (Jones et al., 2007; Kwon et al., 2007). *HillGR5* was clustered with the *Drosophila* saponin receptor *DmelGR28b* (Sang et al., 2019). *HillGR10* were clustered with *Drosophila* sugar receptors (*DmelGR64a*) (Dahanukar et al., 2007). All putative GR-encoding genes were expressed at very low levels, with an average FPKM value of 5.87, except three genes encoding carbon dioxide GRs, which showed higher expression levels with FPKM values 24.49, 10.22, and 9.3, respectively.

### Candidate Sensory Neuron Membrane Proteins (SNMPs)

Two SNMP-encoding unigenes were identified. These two unigenes have full-length ORFs encoding proteins with two TMDs (Supplementary Table S2: Sheet 6). The two *H. illucens* SNMPs were distributed among two different scaffolds. Based on a phylogenetic analysis, all SNMPs were classified into two distinct subfamilies, SNMP1 and SNMP2 (Supplementary Figure S4). *HillSNMP1* was clustered with the SNMP1 subfamily, while *HillSNMP2* clustered with the SNMP2 subfamily. One SNMP unigene, *HillSNMP1*, was expressed at relatively high levels with an FPKM value of 1,338.07 in *H. illucens* antennae (Supplementary Table S2: Sheet 6).

### Tissue- and Sex-Specific Expression

**FIGURE 6:**
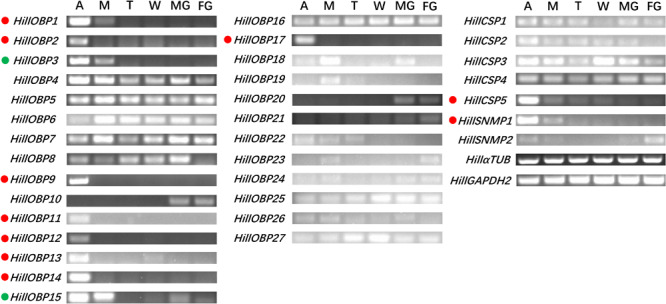
Tissue- specific expression of *HillOBPs*, *HillCSPs*, and *HillSNMPs* as measured by RT-PCR. Genes predominantly expressed in antennae are highlighted with red dots. Genes predominantly expressed in antennae and mouthparts are highlighted with green dots. Control RT-PCR corresponds to α-tubulin and glyceraldehyde-3-phosphate dehydrogenase. A, antennae; M, mouthparts; T, foreleg tarsus; W, wings; MG, male genitals; FG, female genitals.

RT-PCR showed that eight OBP-encoding unigenes (*HillOBP1-2*, *9*, *11-14*, and *17*) were almost exclusively expressed in antennae, while *HillOBP3* and *15* were highly expressed in both antennae and mouthparts (Figure 6). The remaining OBP-encoding unigenes were abundant in multiple tissues. Among the CSP-encoding unigenes, *HillCSP5* was exclusively expressed in antennae, while other *HillCSPs* were present in multiple tissues. In addition, *HillSNMP1* were mainly expressed in antennae.

**FIGURE 7:**
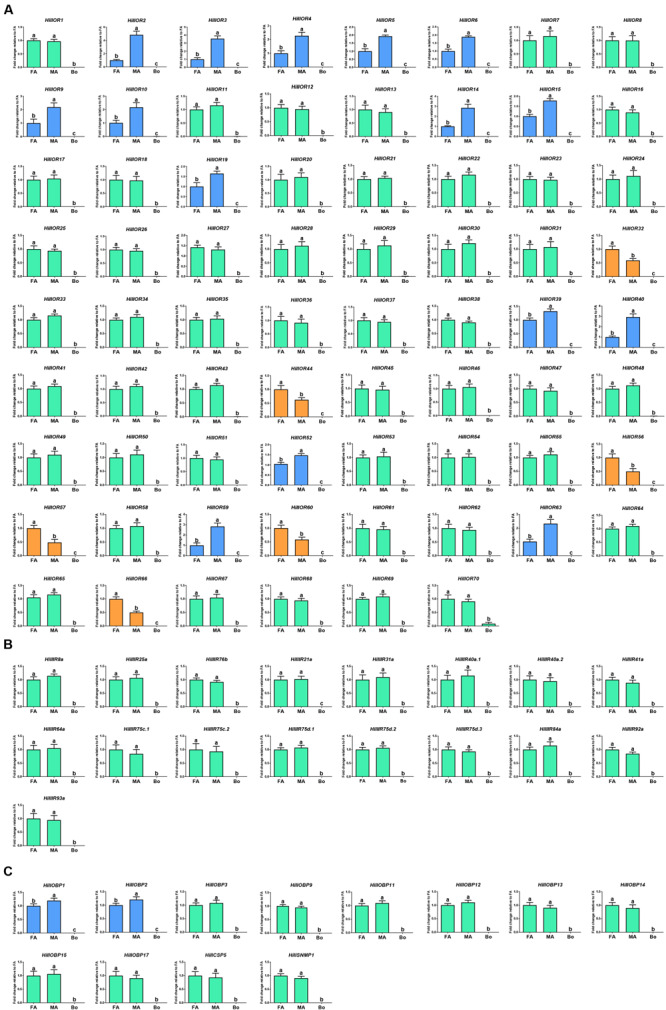
Transcript levels of the olfactory-related genes in different tissues as measured by RT-qPCR. **(A)** ORs. **(B)** Antennal IRs. **(C)** The antennae-enriched candidates (OBPs, CSPs, and SNMPs). FA, female antennae; MA, male antennae; Bo, other body parts. Highlighted histograms: the male-biased ORs (blue); the female-biased ORs (orange); the non-sex-biased ORs (green). Error bars represent standard error. Different small letters on bars indicate significant differences (*p* < 0.05, ANOVA, HSD).

In the OR-encoding unigenes, there were 15 ORs (*HillOR2-6*, *9*, *10*, *14*, *15*, *19*, *39*, *40*, *52*, *59*, and *63*) and six (*HillOR32*, *44*, *56*, *57*, *60*, and *66*) with significantly higher expression in the male and female antennae, respectively (Figure 7A). Among antennal IR-encoding unigenes, all were equally expressed in the male and female antennae (Figure 7B). Among OBP-encoding unigenes, there were two (*HillOBP1* and *2*) with significantly higher expression in males. In addition, *HillCSP5* and *HillSNMP1* were equally expressed in the antennae of both sexes (Figure 7C).

## Discussion

*Hermetia illucens*, a representative species of the Stratiomyidae family, is evolutionally different from many well characterized Dipteran species such as *D. melanogaster*, *M. domestica*, *B. dorsalis*, *C. stygia*, *G. morsitans*, and *M. destructor* (Brammer and von Dohlen, 2007). Homology analyses of the identified *H. illucens* chemosensory genes along with orthologs from other Dipterans show clear separation of the *H. illucens* genes from the ones from other flies (Supplementary Figure S1B). However, *H. illucens* adults exhibit both similarity and differences in mating and oviposition behaviors with *D. melanogaster* and other flies. For example, *H. illucens* males exhibit territorial lekking behavior during mating (Tomberlin and Sheppard, 2001) and can also detect odors released due to the action of bacteria for oviposition (Zheng et al., 2013). Thus, systematic research on chemoreception may provide valuable information on understanding the evolution of the insect olfactory system. *H. illucens* larvae play pivotal roles in both environmental and economic aspects of waste disposal and processing. Artificial release of *H. illucens* adults is a well-known method for waste treatment, but it is difficult to establish populations in the field. Understanding of the molecular recognition mechanisms and the molecular basis for egg-deposition behavior in response to environments has important implications for population establishment after release. With the recent release of the *H. illucens* genome, chemosensory genes have been annotated (Zhan et al., 2020). In this study, all the chemosensory genes identified in the antennal transcriptome of *H. illucens* are present in the genome. This result supports strongly the quality of the transcriptomic assembly.

OBPs identified in *H. illucens* are not very conserved in comparison with those from other Dipteran species. Phylogenetic analysis reveals that only a small subset of OBPs (such as *HillOBP1*, *2*, *3*, *5*, *9*, *11*, and *14*) identified in *H. illucens* have homologs from other Dipterans (Figure 1). Only two types of OBPs are found in *H. illucens*: classic and plus-C. Minus and dimer OBPs which have been reported from other Dipteran species were not found in *H. illucens*. The missing of Minus and dimer OBPs may reflect physiological and evolutionary differences between *H. illucens* and other Dipteran species. Nevertheless, there are some OBPs that are conserved and have orthology relationships with counterparts from other Dipterans. For example, *HillOBP1* and *2* are homolog to *OBP83b* found in *B. dorsalis*, *HillOBP11* is a homolog to *Lush* found in *D. melanogaster*, and *HillOBP9* is a homolog to *DmelOBP59a* found in *D. melanogaster* (Pikielny et al., 1994; Galindo and Smith, 2001; Wu et al., 2015; Sun et al., 2018). Both *OBP83b* and *Lush* have been reported to play roles in sensing semiochemical attractants and the pheromone 11-cis vaccenyl acetate (Xu et al., 2005; Wu et al., 2016). *DmelOBP59a* has been reported with a function in hygroreception (Sun et al., 2018). *HillOBP1, 2*, *9*, and *11* are expressed only in antennae, similar to what has been observed in other Dipterans. OBP genes present in the same genomic cluster show same pattern of expression in antennae. Two pairs of OBPs *HillOBP1*/*2*, *HillOBP12*/*17* are predominantly expressed in antennae and are organized in tandem on scaffold 1521 and 2279, respectively. Based on the phylogeny in Figure 1, it suggest that there has been recent gene duplication. In addition to genes encoding OBPs, a gene encoding a CSP, *HillCSP5*, is also exclusively expressed in antennae. Therefore, *HillOBP1*, *2*, *11*, and *HillCSP5* are likely to play a role in antennal chemical-recognition.

A total of 70 OR unigenes are identified from *H. illucens* antennae, which is a greater number than those identified from the antennae of other Dipterans, such as *B. dorsalis* (60 OR genes identified) (Jin et al., 2017) *C. stygia* (50) (Leitch et al., 2015) *Episyrphus balteatus* (51) and *Eupeodes corollae* (42) (Wang et al., 2017) *D. melanogaster* (39) (Menuz et al., 2014) *Scaeva pyrastri* (38) (Li et al., 2016), and *Chlorops oryzae* (25) (Qiu et al., 2018). The high number of OR-encoding unigenes may be associated with its ability for diverse host-odor detection in *H. illucens*. Surprisingly, except Orco, no OR orthologs to the identified *H. illucens* OR genes are found from other Dipteran species. Apparently, a species-specific expansion has happened to form the 69 OR-encoding genes in *H. illucens* (Figure 3). Gene expansion often reflects the adaptation of a species to its ecological niche. This major OR gene expansion seems to indicate that *H. illucens* require multiple OR genes to detect a diversity of structurally similar odorants in its living habitat. In addition, several ORs within tandem arrays including *HillOR2/3/7/14/23/40/47/55/60*, *HillOR24/29/33/41/49*, and *HillOR13/19/26/31/43*, form the same phylogenetic clade, respectively (Figure 3). It is likely to result from a relatively recent local gene duplication.

ORs expressed predominantly in female antennae are predicted to function in oviposition-related odorant (Pelletier et al., 2010) or male released pheromones detection (Anderson et al., 2009). ORs expressed equally in the male and female antennae are predicted to function in the detection of general odorants such as feeding attractants (Yan et al., 2015). *HillOR32, 44, 56, 57, 60*, and *66* are predominantly expressed in female antennae, and therefore are likely involved in regulation of female-specific behaviors, such as localization of oviposition sites and responses to the pheromones released by males. *HillOR2-6*, *9*, *10*, *14*, *15*, *19*, *39*, *40*, *52*, *59*, and *63* are predominantly expressed in male antennae, and therefore, may be associated with the detection of female sex pheromones. The remaining ORs are roughly equally expressed in both female and male antennae, and therefore, may be involved in general odorant detection (Figure 7).

Seventeen antennal IR genes are identified in this study from *H. illucens*. In *Drosophila*, IR92a, IR84a/8a, IR76b/IR41a, IR75a/IR8a, IR64a/IR8a have been reported to be involved in, respectively, sensing ammonia and amines (Min et al., 2013) phenylacetaldehyde and phenylacetic acid (Grosjean et al., 2011) polyamines (Hussain et al., 2016) acetic acid (Prieto-Godino et al., 2016), and other acids (Ai et al., 2010). IR93a/IR68a/IR40a and IR21a/IR25a have been reported to be responsible for, respectively, temperature and moisture detection (Knecht et al., 2016, 2017) and cool sensing (Ni et al., 2016). IR genes identified here in *H. illucens* show sequence similarity to some of these characterized IRs and they may have similar functions.

Ten genes encoding GRs are identified from this study in *H. illucens*, which is similar to the 12 GR genes reported in the antennae of *D. melanogaster* (Menuz et al., 2014). However, the number of GRs found in *H. illucens* is much fewer than those reported in other Dipteran species, such as *C. stygia* (21 GR genes reported) (Leitch et al., 2015). GRs are known to function as taste and contact receptors and are often involved in host-specific pollination behavior (Jauker et al., 2009; Lucie et al., 2013). Some of the identified GRs from *H. illucens* show orthologous relationship with GRs from other insect species. For example, *HillGR5* is homologous to *DmelGR28b*, a saponin receptor in *D. melanogaster*, and *HillGR10* is homologous to *DmelGR64a*, a sugar receptor. Those GR homologs identified here in *H. illucens* may play similar roles as reported in other insects.

Two SNMP unigenes are identified in this study. Both SNMPs are conserved compared with other holometabolous insect species. SNMP1 is usually expressed in pheromone-sensitive olfactory sensory neurons, and mediates responses to lipid pheromones (Jin et al., 2008; Nichols and Vogt, 2008; Vogt et al., 2009; Gomez-Diaz et al., 2016). In *H. illucens*, SNMP1 is predominantly expressed in antennae, supporting a role of SNMP1 in pheromone detection.

## Data Availability Statement

All data generated during this study are included in this article/Supplementary Material.

## Author Contributions

QX performed the experiments. ZW analyzed the data. QX, ZW, XA, and XZ wrote and revised the manuscript.

## Conflict of Interest

The authors declare that the research was conducted in the absence of any commercial or financial relationships that could be construed as a potential conflict of interest.

## Footnotes

1 https://www.ncbi.nlm.nih.gov/orffinder/

2 http://primer3.ut.ee/

## Supplementary Material

FIGURE S1 Transcriptome overview of *H. illucens* antennae. **(A)** Unigenes annotated in NCBI-nr, NCBI-nt, Swiss-Prot, GO, COG and KEGG databases. **(B)** Species distribution of the top *H. illucens* unigenes best BLASTx hits. BLAST analysis against the non-redundant protein database was performed with a cut-of *E*-value of 10^–5^. **(C)** Gene ontology (GO) term assignment of the *H. illucens* unigenes.

FIGURE S2 Multiple amino acid alignment of *HillOBPs*.

FIGURE S3 Multiple amino alignment of *HillCSPs*.

FIGURE S4 Maximum likelihood of candidate SNMPs from *H. illucens* and other Dipteran insects. Branch support was estimated using 1000 bootstrap replicates, and bootstrap values were displayed with color circles at the branch nodes. The scale bar indicate the expected number of amino acid substitutions per site.

TABLE S1 Chemosensory genes from Dipteran species.

TABLE S2 Candidate chemosensory genes and reference gene of *H. illucens* antennal transcriptome. **Sheet 1**: Unigenes of candidate OBP with gene name, sequence information, best BLASTx hit in NCBI-nr database, predicted domains and FPKM, etc. **Sheet 2**: Unigenes of candidate CSPs. **Sheet 3**: Unigenes of candidate ORs. **Sheet 4**: Unigenes of candidate IRs. **Sheet 5**: Unigenes of candidate GRs. **Sheet 6**: Unigenes of candidate SNMPs. **Sheet 7**: Unigenes of reference genes.

TABLE S3 Amino acid sequences of *H. illucens* and other insect used in the phylogenetic analyses.

TABLE S4 Ranking of the six reference genes of *H. illucens* using Normfinder and BestKeeper in different tissues.

TABLE S5 Primer pairs used for RT-PCR and RT-qPCR.

TABLE S6 Summary of the transcriptome sequencing and assembly process.

TABLE S7 Distribution of unigene length interval in the *H. illucens* antennal transcriptome assembly.

TABLE S8 A list of the expression level of all unigenes in *H. illucens* antennae. Expression level is listed in order from highest to lowest.
