# Supplementary figures and images for: Identification and Expression Profiling of Chemosensory Genes in Hermetia illucens via a Transcriptomic Analysis
Source: Front Physiol. 2020 Jun 19;11:720. doi: 10.3389/fphys.2020.00720 (PMC7325966; doi:10.3389/fphys.2020.00720)

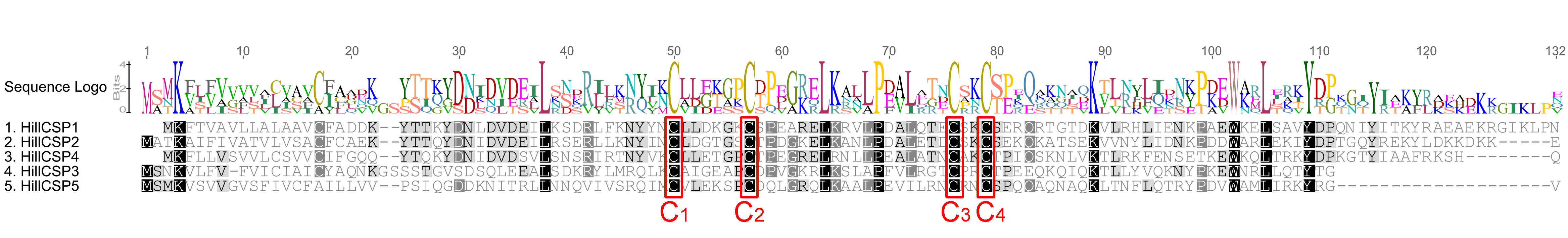

Supplement: FIGURE S3 — Multiple amino alignment of HillCSPs. [file Image_3.TIF]

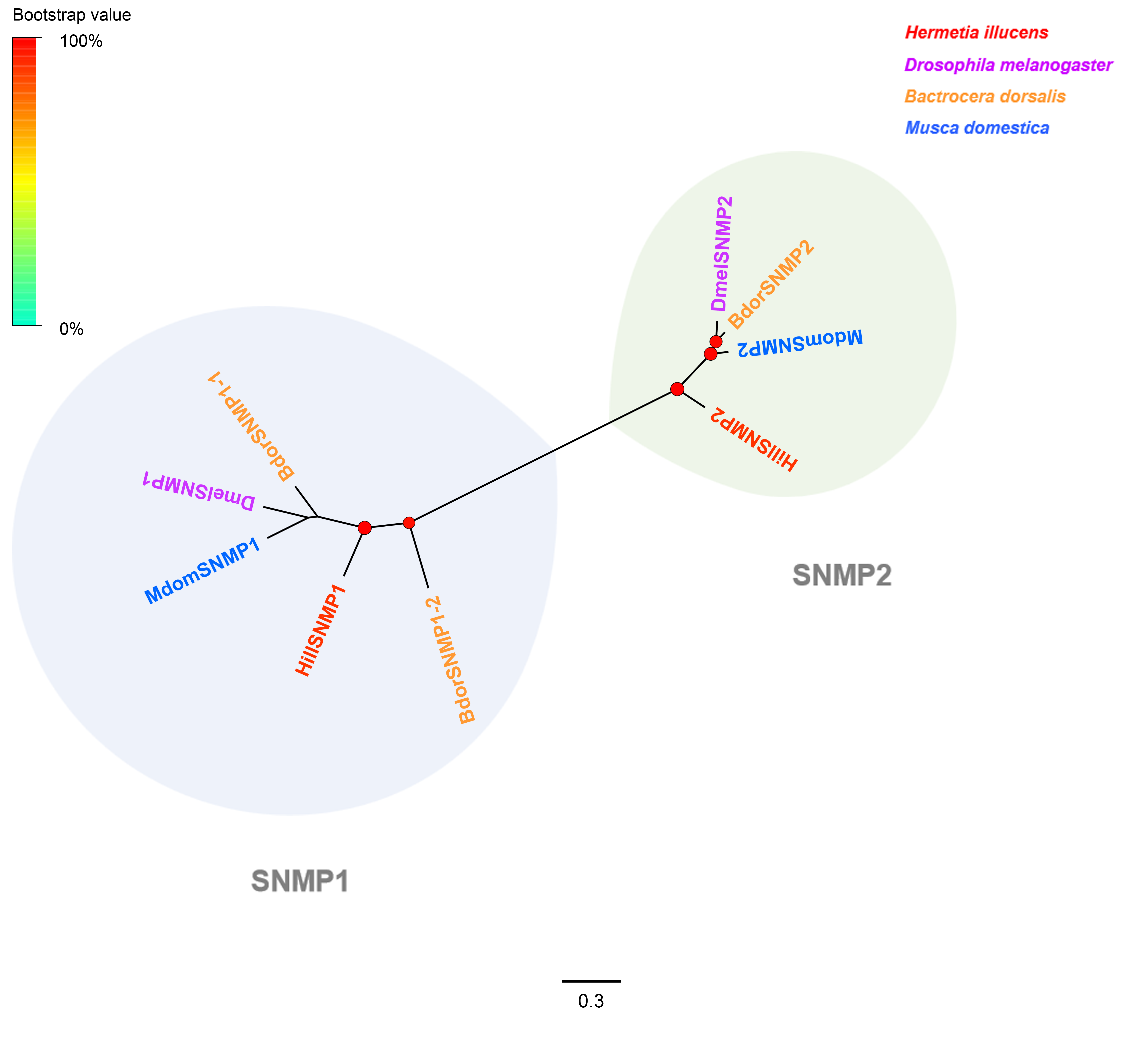

Supplement: FIGURE S4 — Maximum likelihood of candidate SNMPs from H. illucens and other Dipteran insects. Branch support was estimated using 1000 bootstrap replicates, and bootstrap values were displayed with color circles at the branch nodes. The scale bar indicate the expected number of amino acid substitutions per site. [file Image_4.TIF]
